# Effects of different feedback types on information integration in repeated monetary gambles

**DOI:** 10.3389/fpsyg.2014.01597

**Published:** 2015-01-23

**Authors:** Peter Haffke, Ronald Hübner

**Affiliations:** Graduate School of Decision Sciences, Department of Psychology, Universität KonstanzKonstanz, Germany

**Keywords:** risky choice, information integration, feedback, repeated gambles, conditional choice functions (CCFs)

## Abstract

Most models of risky decision making assume that all relevant information is taken into account (e.g., von Neumann and Morgenstern, 1944; Kahneman and Tversky, 1979). However, there are also some models supposing that only part of the information is considered (e.g., Brandstätter et al., 2006; Gigerenzer and Gaissmaier, 2011). To further investigate the amount of information that is usually used for decision making, and how the use depends on feedback, we conducted a series of three experiments in which participants choose between two lotteries and where no feedback, outcome feedback, and error feedback was provided, respectively. The results show that without feedback participants mostly chose the lottery with the higher winning probability, and largely ignored the potential gains. The same results occurred when the outcome of each decision was fed back. Only after presenting error feedback (i.e., signaling whether a choice was optimal or not), participants considered probabilities as well as gains, resulting in more optimal choices. We propose that outcome feedback was ineffective, because of its probabilistic and ambiguous nature. Participants improve information integration only if provided with a consistent and deterministic signal such as error feedback.

## Introduction

Risky choice behavior is often investigated by analyzing how persons choose between different options or lotteries of a monetary gamble. For these structures, which are defined by the possible outcomes (gains or losses) and associated probabilities, the optimal decision can be obtained by calculating the expected value (EV) for each lottery. Consequently, a decision can be considered optimal if the option with the largest EV is chosen.

Soon after the introduction of such choice problems it became clear that people often do not decide “rationally” in this sense of optimality. Therefore, researchers proposed alternative models of human choice behavior. A first idea was that the monetary gains of a gamble do not necessarily represent their subjective value for the decision maker. Accordingly, *utility functions* were introduced (Bernoulli, 1954) that transform monetary gains into utilities, i.e. subjective values reflecting the amount of satisfaction the gains will eventually produce. By substituting the monetary values of a gamble by their utilities, as assumed in the *Expected Utility* (EU) theory (von Neumann and Morgenstern, 1944), the expected utility can be computed for each option, and a decision is considered as optimal, if the option with the highest result was chosen. However, even EU theory could not satisfactorily account for some aspects of human choice behavior. Therefore, in their *Prospect Theory* (PT), Kahneman and Tversky (1979) assumed, among others, that the probabilities within a gamble have also to be transformed to represent systematic subjective distortions (e.g., underestimation of small probabilities).

Obviously, these models assume that all relevant information for finding an optimal choice is available. Accordingly, they were mostly tested in so-called *description-based* decision studies, where fully described gambles are presented once (e.g., Hertwig et al., 2004). For many decisions, however, one rarely knows all relevant facts (e.g., Simon, 1955). Therefore, *experience-based* decision studies have also been conducted, where gambles, like the *Iowa Gambling Task* (IGT; Bechara et al., 1994), are described only partially but administered repeatedly. Obviously, in these studies part of the participants' task is to learn and/or infer the defining probabilities and values of a gamble from the feedback of gains and losses.

However, even if the information for an optimal decision is available, as in description-based studies, participants do not necessarily process all the relevant data. Gigerenzer and colleagues, for instance, have shown that decisions are often based on heuristics that take only a fraction of the available information into account (e.g., Brandstätter et al., 2006; Gigerenzer and Gaissmaier, 2011). Nevertheless, it is conceivable that, if fully described gambles are processed repeatedly, information processing and the applied heuristics change with experience. Unfortunately, little is known in this respect as the focus was on either description-based gambles (e.g., Brandstätter et al., 2006; Glöckner and Betsch, 2008; Rieskamp, 2008; Fiedler and Glöckner, 2012), experience-based gambles (e.g., Lejuez et al., 2002; Barron and Erev, 2003; Hertwig et al., 2004), or their comparison (e.g., Hertwig et al., 2004; Camilleri and Newell, 2011; Glöckner et al., 2012). Therefore, the aim of the present study was to investigate gambles that combine both characteristics. One question was which decision strategies are applied. In most studies, participants were not informed about the outcome of their single choices, which presumably prevented learning. However, if this information is provided, participants may be encouraged to test different strategies to figure out their effectiveness and eventually maintain the most successful one instead of continuously applying their initially preferred strategy. Because strategy evaluation largely depends on feedback, a further question was, to what extent strategies and choices in repeated gambles depend on the type of the provided information. Yechiam and Busemeyer (2006), for example, found that choices were generally less risky when participants were informed about the outcome after each choice. However, different feedback types vary with respect to their information content so that they presumably also affect the learning of choice behavior differently. In the present study, we tested how outcome feedback and normative error feedback influence choices compared to when feedback is absent.

**Figure 1:**
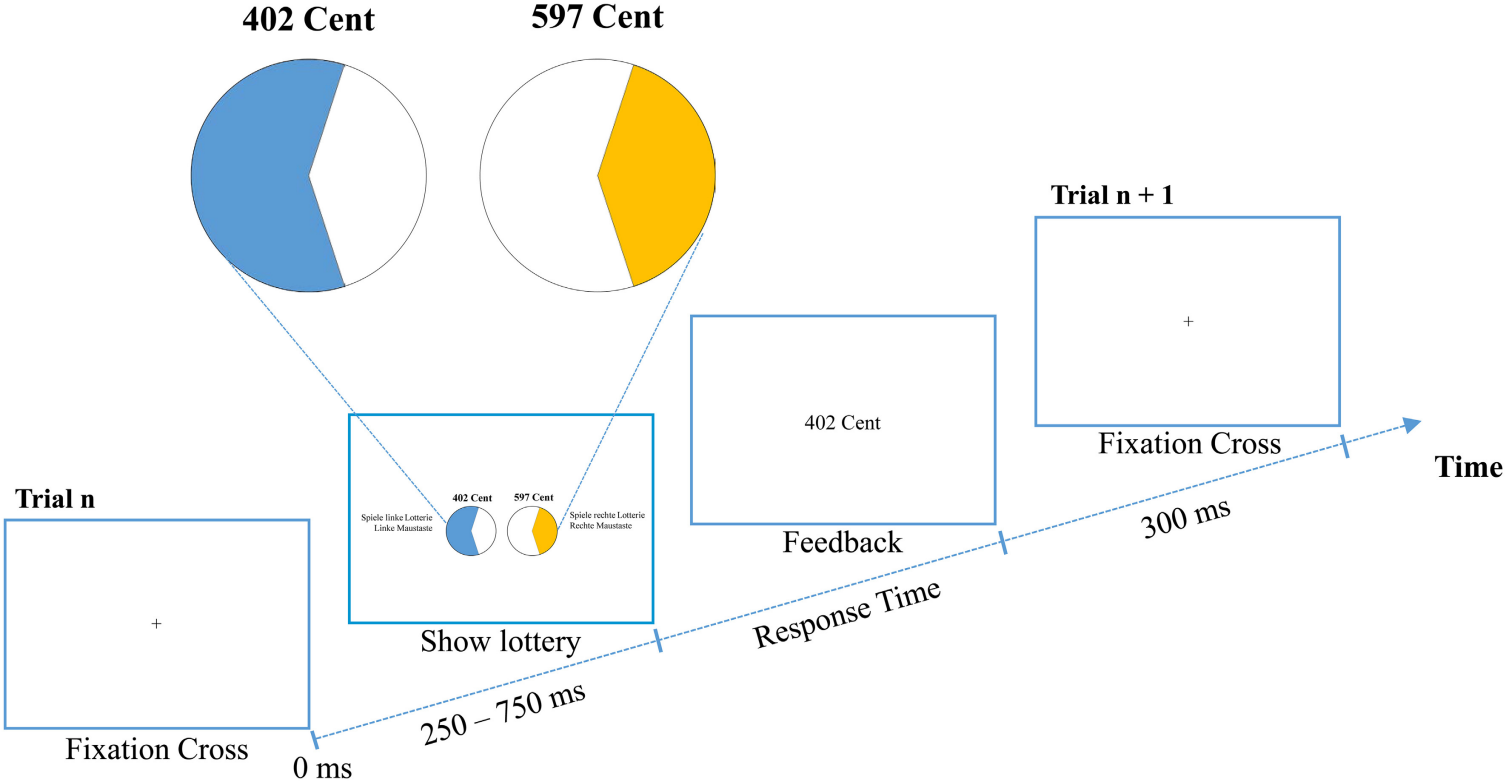
**Experimental procedure of the Wheel of Fortune task with outcome feedback as used in Experiment 2**. Participants had to decide whether they wanted to play the lottery with the higher winning probability (left) or the one with the higher potential gain (right).

For investigating choice performance in the present study, we applied a specific version of the *Wheel of Fortune* task (WOF; Ernst et al., 2004; Smith et al., 2009). In this computerized gamble participants had to choose one of two lotteries, A and B. In each lottery they could win a certain amount of money *x* with probability *p* or nothing (*x* = 0) with probability 1 – *p*. Moreover, the winning probabilities of the two competing lotteries added up to 1 (i.e., *p_A_* = 1 – *p_B_*). The probabilities of each lottery were presented as pie charts, and the gains as numbers above the corresponding pies (see Figure 1). Thus, because all relevant information was available, according to the PT, EU, and EV theories, the lottery with the larger attractiveness, expected utility, or expected value should be chosen, respectively. However, there are also heuristics that use only partial information. For instance, the *Most-Likely* (ML) heuristic demands to select the lottery with the larger winning probability. With respect to the gambles used in our experiments, this rule is equivalent to the *Priority Heuristic* (PH; Brandstätter et al., 2006). Alternatively, one can chose the lottery with the larger gain, as stated by the *Maximax* (MM) heuristic.

The aim of the present study was to examine how well these different theories and heuristics account for human choice performance. For this objective, two types of gambles were constructed and presented equally often. In *pro-win* gambles, the EV-optimal choice (optimal according to EV theory) was associated with the higher winning probability, whereas in *pro-gain* gambles it corresponded to the higher amount of money (see Table 1). Consequently, a person who applies the ML heuristic (i.e., chooses the lottery with the higher winning probability), performs EV-optimally in *pro-win* gambles, but not in *pro-gain* gambles. In contrast, for persons applying the MM heuristic, the opposite would be the case. Consequently, only if probabilities and gains are combined in some beneficial way, choices can be optimal in more than 50% of the trials.

**Table 1:** **Overview of the composition of lotteries regarding the presented lottery information as indicator for optimal and non-optimal choices (gamble type)**.

**Type of gamble**	Optimal choice^*^		**Suboptimal choice**	
	**Probability**	**Gain**	**Probability**	**Gain**
*pro-win*	high	small	low	large
*pro-gain*	low	large	high	small

* *Choosing the lottery with the higher expected value is defined as optimal choice*.

The two types of gambles also served our goal to examine the dynamics of choice processes. Obviously, the strategies differ in complexity, which suggests that their application differs in mental effort. ML and MM are relatively easy to perform and, therefore, might be executed relatively automatically, whereas EV and PT are based on calculations that require controlled and effortful mental processes. Thus, first of all, we expected that choices based on ML and MM are faster than those relying on EV and PT. Moreover, we hypothesized that individuals apply different strategies across trials. For instance, it is conceivable that automatic and controlled processes compete in a race, as assumed by dual-process models (e.g., Hübner et al., 2010; Mukherjee, 2010). Thus, even if persons intend to integrate the provided information, on some trials, the required slow computational processes might be superseded by a fast automatic process that chooses, for instance, the lottery with the higher probability of winning. A gain-domain specific overweighting of higher probabilities over higher outcomes for lotteries with the same EV has already been observed and called *risk-aversion* (Tversky and Kahneman, 1981), *p*-*dominance*, or *probability-dominance* (Fiedler and Unkelbach, 2011). Because this dominance is presumably most influential for fast responses, it needs to be suppressed by controlled processes to allow for an integration of values. We therefore expected that choice performance improves with an increasing response time, at least in *pro-gain* gambles in which information integration is beneficial. It has already been proposed that decision makers adaptively choose strategies from a toolbox (Payne et al., 1993). A race between strategies could also explain why studies in which individuals are classified according to their applied strategy often identify more than one strategy for a single person (e.g., Glöckner, 2009; Davis-Stober and Brown, 2011).

To see whether the observed response times (RTs) indicate a mixture of automatic and controlled processes, we considered *conditional choice functions* (CCFs). These functions are adapted versions of conditional accuracy functions, which have been applied to analyze perceptual decisions (e.g., Gratton et al., 1992; Hübner and Töbel, 2012). CCFs provide choice proportions as function of RT, and can, as we will demonstrate, give useful insights in the domain of risky choices. For example, if the same strategy is used throughout an experiment, and the speed of the corresponding processes merely varies randomly across trials, then the CCFs should be flat. However, if fast choices are caused by automatic heuristics and slow ones by complex but favorable computations, then the proportion of optimal choices should systematically increase with RT.

Our ideas were tested in three experiments, which differed with respect to the type of feedback that was provided. After observing performance without any feedback (Experiment 1), we provided outcome feedback (Experiment 2) and error feedback (Experiment 3). Additionally, to analyze mean performance and changes in choice behavior within the experiments, we also fitted different decision models to the data and compared their overall performance. Finally, CCFs were computed and analyzed.

## Experiment 1

Our first experiment served for collecting baseline choice data in a condition without any feedback. The absence of feedback is common in risky-choice experiments, especially in those with a one-shot paper-pencil procedure. Here, a choice between lotteries was required repeatedly. Apart from fitting and comparing the performance of different choice strategies, the central focus was on gathering information about the time course of how the presented lottery information is taken into account.

### Methods

#### Participants

A total of 17 participants (12 female), aged between 19 and 58 years (*M* = 24.5, *SD* = 8.9), from the Universität Konstanz, participated in the experiment. Participants, recruited via our laboratory's participant database, received either course credit or money at the end of the experiment. They were told that, in addition to a base payment of €5, they could win a certain proportion of €5, depending on their decisions (i.e., the total proportion of money actually won across all trials from the maximum possible amount)^1^.

#### Material and procedure

As task served a specific version of the *Wheel of Fortune* task (WOF; Ernst et al., 2004; Smith et al., 2009). Each gamble had one of two combinations of winning probabilities: 60:40, and 80:20. The first combination, for example, means that one lottery had a 60% chance of winning a certain amount of money and a 40% chance of winning nothing. The competing lottery had a 40% chance of winning a certain amount of money and a 60% chance of winning nothing. The two probabilities were represented by two pie charts. As shown in Figure 1, the colored (blue and orange) portions of the pie reflected the winning probabilities, where blue always indicated the higher probability. The white areas represented the probabilities of winning nothing.

The gains for each lottery ranged from 1 to 600 *Cent* (*Eurocent*). They were randomly selected, but restricted in two ways: First, for each gamble, the difference in gain between the two lotteries could either be *50* or *200 Cent*, with a ±10 Cent jitter. By jittering the values, variability was increased in order to minimize learning effects through recognition of specific pairs, and also allowed to test the influence of the magnitude of the value difference. Second, probability-value pairs had to be in line with our manipulation of gamble type, as explained below.

In the original version of the WOF (Ernst et al., 2004; Smith et al., 2009), the option with the highest winning probability had an overall advantage with respect to choosing EV-optimally. The gambles in our experiments, however, can be categorized into two types. For *pro-win* gambles, the lottery with the higher winning probability represents the optimal choice according to EV theory, whereas for *pro-gain* gambles the lottery with the higher gain is optimal. An overview of these configurations can be found in Table 1. It is noteworthy that we omitted lotteries where both the higher probability and the higher gain indicated EV-optimality.

Participants had to choose the left or right lottery as quickly as possible by pressing the left or right mouse key, respectively. The left/right position of the lotteries was randomized across trials. Each trial started with the presentation of all information (see Figure 1). After each choice, another gamble was presented. Participants had one training block to familiarize with the mode of presentation.

Altogether, the experiment comprised a 2 (80:20 vs. 60:40) × 2 (50 vs. 200 *Cent*) × 2 (*pro-win* vs. *pro-gain*) within-participant design. For each of the 8 condition there were 120 trails, resulting in 960 trials (divided in 40 blocks of 24 randomized trials). Participants were not informed about the number of trials to avoid riskier choices at the end of the experiment. However, they were informed about the length of the study.

#### Analysis of strategy fit

To assess whether the observed choice proportions are in line with a specific strategy or heuristic, we compared five prominent choice strategies (of which two yielded the same predictions). The strategies differ with respect to the extent to which the available information is used.

- *Most-Likely / Priority Heuristic* (ML/PH): For both strategies, the lottery with the higher winning probability has to be chosen. According to the *Most-Likely* heuristic, only the highest winning probabilities are compared. The *Priority Heuristic* assumes that choices are made by sequentially comparing minimum gains, minimum probabilities and maximum gains. The examination of a gamble is stopped if, for example, minimum gains differ by 1/10 of the maximum gain, otherwise the next aspect of the gambles is examined. For the gambles in this experiment ML and PH predict the same choices^2^.
- *Maximax* (MM): The lottery with the larger gain has to be chosen, irrespective of the winning probabilities.
- *Expected Value theory* (EV): For each lottery, the expected value (probability × gain) is computed and the lottery with the higher EV is chosen.
- *Cumulative Prospect Theory* (CPT): The lottery with the higher attractiveness according to CPT is chosen. How attractiveness is calculated and how the required parameters were estimated for every subject using a probabilistic choice rule is described in next section. The averaged parameter estimates for all experiments can be found in Table 2. For each of the 960 lotteries, predictions were computed.

**Table 2:** **Averaged parameters for Cumulative Prospect Theory (CPT)**.

**CPT Parameter**	**Experiment 1 No feedback**	**Experiment 2 Outcome feedback**	**Experiment 3 Error feedback**
α	0.68 (0.35)	0.32 (0.32)	0.93 (0.24)
γ	1.40 (0.21)	1.29 (0.44)	1.40 (0.36)
δ	0.36 (0.27)	0.97 (1.08)	0.36 (0.11)
φ	0.69 (0.78)	2.23 (2.77)	0.01 (0)

*Values show the mean parameters with standard deviations in brackets*.

#### Parameter estimation of cumulative prospect theory

According to Cumulative Prospect Theory (CPT; Tversky and Kahneman, 1992), the subjective value *V* of a lottery A is defined as,

(1) V (A)=v (x) · w (p),

where the value function *v* characterizes the subjective value of a single lottery's gain *x*, and the probability weighting function *w* denotes the transformation of the corresponding probabilities *p*. In the present study, a lottery consisted of two probability-gain pairs. The potential gain of one pair was always zero. Thus, one probability-gain pair was only needed for the calculation of *V*(*A*).

As value function, we used the function proposed by Tversky and Kahneman (1992),

(2) $$v\;\left(x\right)=x^{\alpha }\text{ if }x\geq 0,$$

where α determines the curvature of the value function. A value of α = 1 would indicate that subjective and objective values are identical, whereas α < 1 indicates decreasing subjective values with increasing objective values.

We furthermore implemented a two-parameter probability weighting function proposed by Gonzalez and Wu (1999),

(3) $$w\;\left(p\right)=\frac{\delta p^{\gamma }}{\delta p^{\gamma }+{\left(1-p\right)}^{\gamma }}\text{ if x}\geq \text{0},$$

where γ controls the curvature, with γ < 1 indicating an overweighting of small probabilities. The parameter δ denotes the elevation of the function, and is interpreted as characterizing the attractiveness of a lottery (Glöckner and Pachur, 2012).

To determine the probability of choosing Lottery A over Lottery B, we used the exponential version of Luce's choice rule^3^,

(4) $$p\left(A,B\right)=\frac{e^{\varphi V\left(A\right)}}{e^{\varphi V\left(A\right)}+e^{\varphi V\left(B\right)}},$$

where *V* denotes the subjective value of the entire Lottery A or B, and φ describes the sensitivity of how the model reacts to differences in-between the subjective values of the two lotteries. A large φ indicates that the choice probabilities are a function of the lotteries' subjective value difference, rather than based on probabilistic choices (e.g., Rieskamp, 2008).

As goodness-of-fit measure we used the *G*^2^ statistic (e.g., Sokal and Rohlf, 1994),

(5) $$G^{2}=-2\sum_{i=1}^{n}\text{ln}[f_{i}\left(y|\;\theta \right)],$$

where *n* denotes the total number of lottery choices, and *f_i_*(*y*|θ) represents the probability of choosing a lottery *y* given parameter set θ. If Lottery A or B was chosen, then *f_i_*(*y*|θ) = *p_i_*(*A*, *B*), or *f_i_*(*y*|θ) = 1 − *p_i_*(*A*, *B*), respectively. Following suggestions of Rieskamp (2008), choice probabilities were truncated to a minimum of 0.01 and a maximum of 0.99.

CPT parameters were restricted to 0 < α ≤ 1, 0 < γ ≤ 1.5, 0 < δ ≤ 4, and 0 < φ ≤ 10. Usually, γ is not allowed to be larger than 1. However, experienced-based decisions are typically characterized by an underestimation of small probabilities, which can be reflected by γ > 1.

To derive the set of best fitting parameters, we used the statistical software R (R Development Core Team, 2010). We first applied a grid search within entire parameter space in steps of 0.1 to derive appropriate starting values for each participant. We then used the optimization function *optim*, with the L-BFG-S method^4^ to obtain a set of best fitting parameter values for each participants by minimizing *G*^2^.

#### Conditional choices functions

To examine how participants' choice behavior varies with response time Conditional Choice Functions (CCFs) were constructed for each experimental condition and participant by sorting the corresponding data into five 20% bins. We then computed the proportion of optimal choices (according to EV theory) and the mean RT for each bin. The resulting values were then averaged across participants in order to obtain a group distribution (for an evaluation of this method, see Rouder and Speckman, 2004).

### Results

All analyses were conducted with R (R Development Core Team, 2010) and visualized using the package *ggplot2* (Wickham, 2009).

Choices with RTs smaller than 200 ms and larger than 3000 ms were considered as outliers and excluded from analysis (<0.6% of all data).

#### Choice behavior

The mean choice proportions show that the lottery with the higher winning probability was chosen on 70.3% (*SD* = 45.8%) of the trials. For *pro-win* gamble this lottery was chosen more frequently (*M* = 82.5%, *SD* = 38.0) than for *pro-gain* gambles (*M* = 57.5%, *SD* = 49.4%). A repeated-measures ANOVA revealed that the factor *gamble type* was significant, *F*(1, 16) = 38.60, *p* < 0.001, η_*G*^2^_ = 0.707.

#### Block by block learning

**Figure 2:**
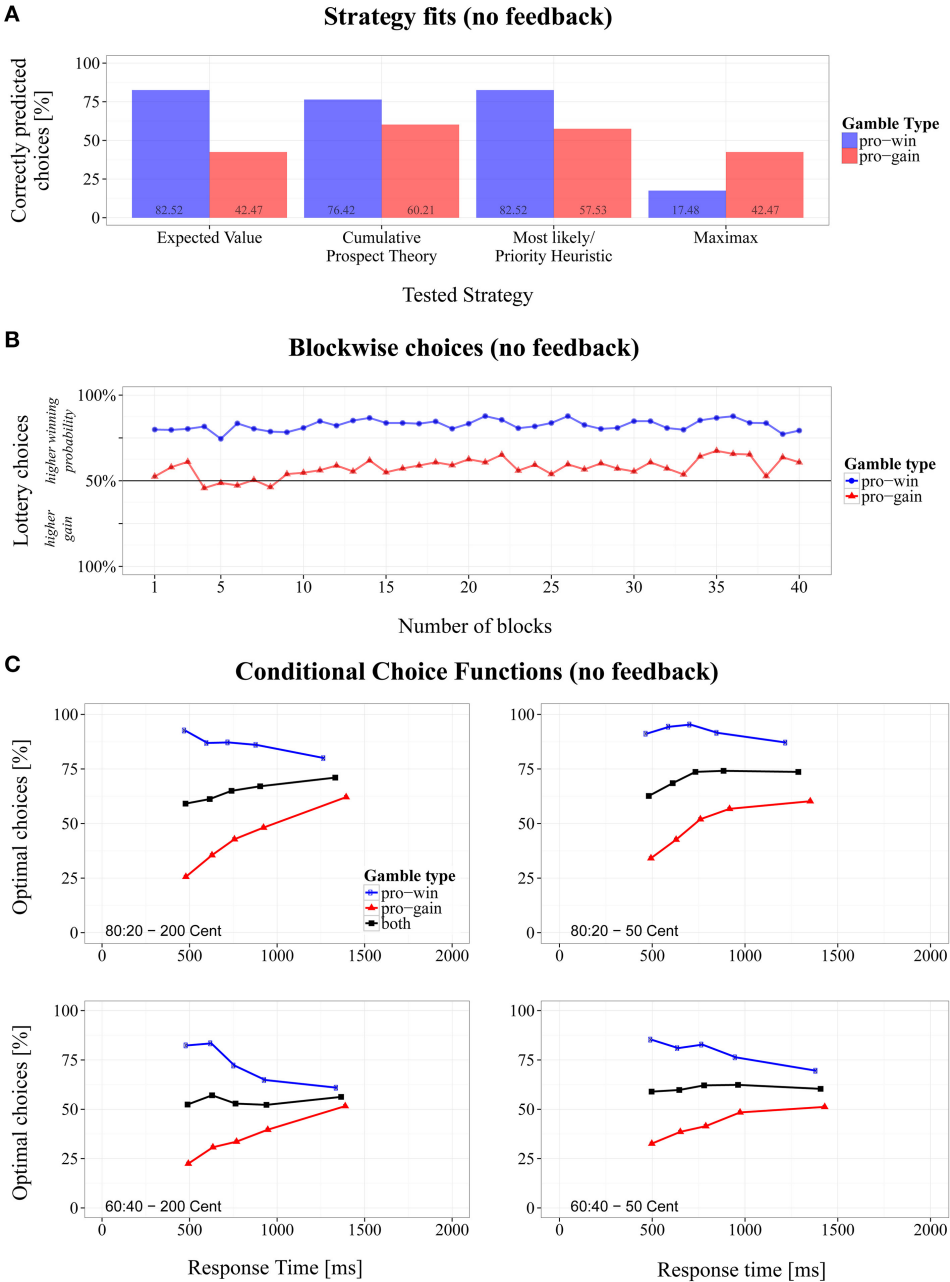
**Overview of the results for the modified Wheel of Fortune task without feedback. (A)** Proportions of correctly predicted choices for *pro-win* and *pro-gain* gambles across different strategies, **(B)** changes of choice proportions across 40 experiment blocks, and **(C)** conditional choice functions (CCFs).

To test whether choice proportions changed across experimental blocks, we subjected the data to a 2 (gamble type) × 40 (block number) repeated-measures ANOVA. No significant effects involving block number were present, suggesting stability over the time course of the experiment (see Figure 2B).

#### Strategy fits

Using the parameter estimates obtained from model fitting, CPT predicted that, on average, lotteries with higher winning probabilities are chosen in 73.4% of the trials. Other strategies predicted overall proportions of either 0% (MM), 50% (EV), or 100% (ML/PH) choices for the same lotteries. As a goodness-of-fit measure, we computed the proportion of correct predictions based on single decisions for each strategy. The strategy with the overall highest fit to the observed data was ML/PH (*M* = 70.0%, *SD* = 45.8%), followed by CPT (*M* = 68.3%, *SD* = 46.5%), EV (*M* = 62.5%, *SD* = 48.4%), and MM (*M* = 30.0%, *SD* = 45.8%). However, paired *t*-tests revealed no significant differences between the fits of EV, CPT, and ML/PH (see Table 3).

**Table 3:** **Paired comparisons of the overall proportion of correctly predicted choices within the three experiments, with t-statistic and effect size Cohen's *d***.

	**No feedback**				**Outcome feedback**				**Error feedback**			
	***t***	***df***	***p***	***d***	***t***	***df***	***p***	***d***	***t***	***df***	***p***	***d***
EV vs. CPT	−1.32	16	0.207	−0.45	−3.30	18	^**^	−1.07	17.49	16	^***^	6.18
EV vs. ML/PH	−1.26	16	0.226	−0.43	−3.00	18	^**^	−0.97	12.60	16	^***^	4.46
EV vs. MM	9.39	16	^***^	3.22	8.76	18	^***^	2.84	12.60	16	^***^	4.45
CPT vs. ML/PH	−0.89	16	0.384	−0.31	−1.80	18	0.088	−0.59	6.51	16	^***^	2.30
CPT vs. MM	5.26	16	^***^	1.81	6.52	18	^***^	2.12	4.09	16	^***^	1.45
ML/PH vs. MM	4.52	16	^***^	1.55	5.56	18	^***^	1.81	0.57	16	0.579	0.2

EV: Expected value; CPT: Cumulative Prospect Theory; ML/PH: Most-Likely/Priority Heuristic; MM: Maximax;

** p < 0.01;

*** *p < 0.001; df: degrees of freedom*.

As Figure 2A shows, the fit was not equally well between gamble types. Except for MM, the strategies fared better in predicting *pro-win* gambles than *pro-gain* gambles. The differences between gamble types were significant for every strategy, paired |*t*s| > 4.51, *p*s <. 001, and Cohen's |*d*s| > 1.55^5^.

#### Conditional choices

Figure 2C shows the CCFs for the two gamble types in the different conditions. As can be seen, for fast responses more EV-optimal choices were made in *pro-win* gambles (blue line), compared to *pro-gain* gambles (red line). This indicates that spontaneously the lottery with the higher winning probability was chosen. With an increasing RT, however, the proportions changed. Whereas the proportion of EV-optimal choices decreased for *pro-win* gambles, it increased for *pro-gain* gambles.

The black lines in Figure 2C represent the average CCFs, which indicate whether the overall performance increased with RT. Linear regression coefficients were computed for each participant and average CCF. The slopes indicate whether the proportion of EV-optimal choices increased with RT in steps of 1 ms (positive slopes), decreased with RT (negative slopes) or remained constant (slopes close to zero). They were subjected to a 2 (probabilities: 80:20, 60:40) × 2 (gain differences: 200, 50) repeated-measures ANOVA.

The analysis revealed an overall increase of optimal responses as the intercept term indicated a significant deviation of the slopes' grand mean (*M* = 0.008, *SD* = 0.037) from zero, with *F*(1, 16) = 9.41, *p* < 0.01, η_*G*^2^_ = 0.232. The base level of optimal responses, that is the averaged point where CCF regression lines intersect with the y-axis at 0 ms, was at 56.9%. In addition, we found a main effect of probability with *F*(1, 16) = 11.03, *p* < 0.01, η_*G*^2^_ = 0.114 (*b*_80:20_ = 0.013 and *b*_60:40_ = 0.003). As can also be seen in Figure 2C, the average proportion of optimal choices increased substantially with RT in *80:20* gambles, but only weakly improved in *60:40* gambles. There were no further significant results.

### Discussion

The results clearly show that participants performed nearly optimally in *pro-win* gambles, but chose suboptimally in *pro-gain* conditions. This indicates that participants based their decisions mostly on partial information. More specifically, they largely neglected the monetary outcomes and preferred the lottery with a higher winning probability. This conclusion is also supported by results obtained from comparing different decision strategies with the observed choice behavior. The ML/PH strategy, according to which the lotteries with the higher chance of winning should always be chosen, explained our results better than the other strategies. The match was even better for *pro-win* gambles, which suggests that these gambles further encouraged the use of such a strategy. CPT did equally well in predicting the choices. In this model probability-dominance was reflected by a rather flat value function (see parameter estimations in Table 2), indicating that choices were mainly driven by probabilities.

By comparing decision strategies with choice proportions, one assumes that participants always use the same strategy throughout the experiment. However, it is reasonable to assume that several strategies compete for execution and that, therefore, the observed performance reflects a mixture of applied strategies. Furthermore, if simple but fast strategies compete with more optimal but slow ones, then this should be reflected by the CCFs. Indeed, as can be seen in Figure 2C, the proportion of optimal choices changed substantially with RT. They decreased for *pro-win* gambles but increased for *pro-gain* ones. This indicates that fast choices relied more on simple strategies that take only partial information into account, whereas slow choices were based on more information. Specifically, the CCFs suggest that fast responses resulted from the application of ML/PH, i.e., from simply choosing the lottery with the higher winning probability. For slower responses more or other information was taken into account. The fact that the overall performance increased with RT indicates that the participants did not only switch from the ML/PH to the MM strategy, because in this case overall performance would have remained constant with RT. Rather, the increase in performance signals that slower responses were indeed based on some integration of probability and gain information, which was particularly the case for *80:20* gambles. That lotteries with a higher chances of winning are generally preferred is already known (e.g., Kahneman and Tversky, 1984). However, up to now, it has not been shown that this preference declines with the duration of processing (but see Dambacher et al., unpublished manuscript).

Although repeated choice performance improved for slow responses, it was still far from optimal. One reason could have been that no feedback was provided. Without this information the participants were obviously not able to adjust their behavior toward optimal performance and simply stuck to their initial choices preference. To see whether feedback helps to improve performance, we conducted a further experiment.

## Experiment 2

This experiment was similar to our first one, except that feedback was provided. Specifically, after each choice, the chosen lottery was played by the computer and the respective outcome (*x* Cent or nothing) was presented on the display. If participants can use this information to improve their choice strategy, then performance should be better than in our first experiment. Moreover, due to learning, performance should now improve during the experimental session. To see whether this is the case, and if so, how quickly learning takes place, we again examined how the proportion of optimal choices varied across the experimental blocks. Finally, learning could produce a generally increased mean RT, because more time is spent for information integration. In the CCFs this should be reflected by a shift to longer RTs and/or flat curves, if a single strategy has been adopted.

### Methods

#### Participants

Nineteen participants (16 female, 3 male; aged between 18 and 45 years, *M* = 22.5, *SD* = 6.3) took part in the experiment. All were students from the Universität Konstanz, and did not participate in the previous experiment. They received either course credit or monetary incentives for their participation, with the same payment structure as in the previous experiment (see Footnote 1).

#### Material and procedure

We used the same procedure as in Experiment 1, except that, after each choice, the chosen lottery was played by the computer and the resulting outcome was presented at the center of the screen. 300 ms later the next gamble started (see Figure 1).

### Results

Responses faster than 200 ms and slower than 3000 ms were considered as outliers and excluded from analysis (<0.6% of all data).

#### Choice behavior

Across all trials, lotteries with a higher probability of winning were chosen in 73.0% (*SD* = 44.2%) of the cases. This proportion was higher for *pro-win* gambles (*M* = 80.5%, *SD* = 39.6%) than for *pro-gain* gambles (*M* = 65.4%, *SD* = 47.6%). A repeated-measures ANOVA indicates that this difference of gamble types was significant, with *F*(1, 18) = 26.58, *p* < 0.001, η_*G*^2^_ = 0.596.

#### Block by block learning

**Figure 3:**
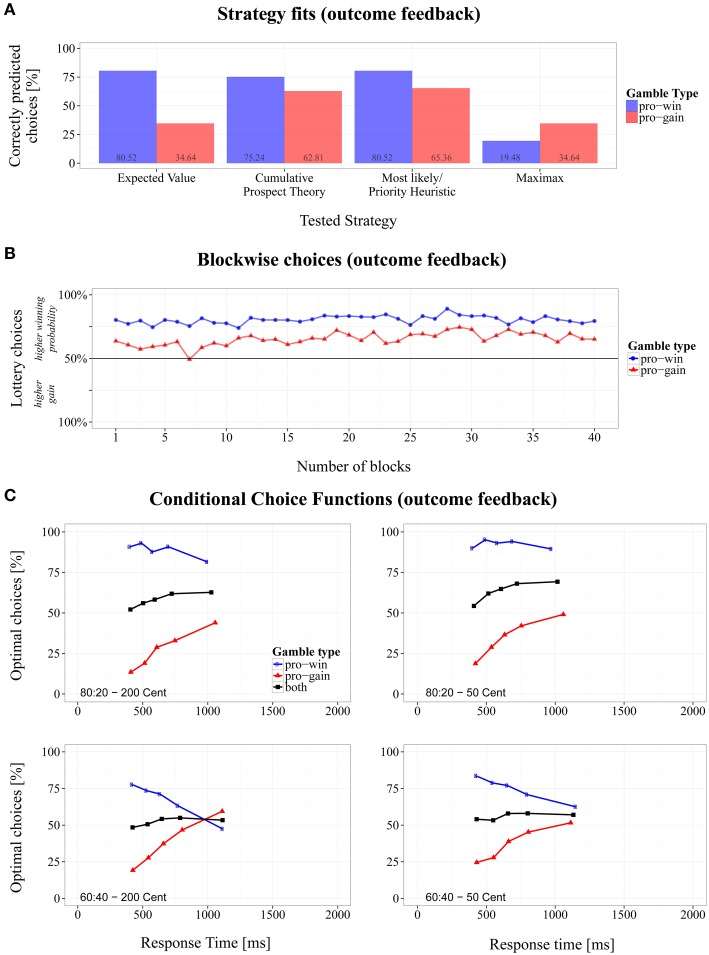
**Overview of the results for the modified Wheel of Fortune task with outcome feedback. (A)** Proportions of correctly predicted choices for *pro-win* and *pro-gain* gambles across different strategies, **(B)** changes of choice proportions across 40 experiment blocks, and **(C)** conditional choice functions (CCFs).

To test whether the choice proportions changed during the session, we extended the repeated-measures ANOVA by the factor *block number* (1 – 40), which revealed a significant interaction, *F*(39, 702) = 1.49, *p* < 0.05, η_*G*^2^_ = 0.020. As Figure 3B shows, choice proportions for the lottery with the higher winning probability increased slightly stronger in *pro-gain* gambles, compared to the relatively constant proportions in *pro-win* gambles. However, the data do not suggest a systematic increase of optimal performance with experience, because this should have resulted in an increasing number of choices for the lottery with higher gain in *pro-gain* gambles.

#### Strategy fits

Figure 3A shows the fit of the different choice strategies. The best fit with the observed performance was obtained for ML/PH (*M* = 73.0%, *SD* = 44.2%) closely followed by the CPT (*M* = 69.6%, *SD* = 46.0%), EV (*M* = 57.6%, *SD* = 49.4%), and MM (*M* = 27.1%, *SD* = 44.2%). Apart from ML/PH and CPT, the overall fit significantly differed between the strategies (see Table 3).

As in Experiment 1, differences for the gambles types could be observed. The strategy fits were better in *pro-win* gambles compared to *pro-gain* gambles, except for MM. The differences between gambles types were significant for each strategy, paired |*t*s| > 5.17, *p*s < 0.001, and Cohen's |*d*s| > 1.68.

#### Conditional choices

A 2 (probabilities) × 2 (gain difference) repeated-measures ANOVA of the CCF slopes revealed a significant intercept, *F*(1, 18) = 21.40, *p* < 0.001, η_*G*^2^_ = 0.333. The positive grand mean of slopes (*M* = 0.011, *SD* = 0.057) indicates an overall increase of optimal responses with RT. The base level of optimal responses was at 49.1%. A significant main effect also occurred for probabilities, *F*(1, 18) = 6.78, *p* < 0.05, η_*G*^2^_ = 0.093, (mean slopes: *b*_80:20_ = 0.016, and *b*_60:40_ = 0.006). As Figure 3Cs shows, for *80:20* gambles more optimal choices were made as RT increased, whereas the increase was only weakly the case in *60:40* gambles. The latter was mainly driven by a strong decline of optimal responses for *pro-win* gambles with 60:40 lotteries.

#### Comparison with Experiment 1

In order to test for differences between Experiments 1 and 2, we compared participants' mean slopes and mean intercepts. For the mean slopes (Exp. 1: 0.008; Exp. 2: 0.011), there was no significant difference, paired *t*(33.3) = −0.85, *p* = 0.401, Cohen's *d* = −0.28. The intercepts (Exp. 1: 56.9; Exp. 2: 49.1), however, did significantly differ, paired *t*(22.8) = 2.10, *p* < 0.05, Cohen's *d* = 0.70. CCFs were highly similar to those in Experiment 1. However, choices for lotteries with high winning probabilities in *pro-gain* gambles were more pronounced in very fast RT bins. This did not affect slope differences but resulted in an overall shift of the intercept toward less optimal responses.

### Discussion

In this experiment outcome feedback was provided after each choice. Surprisingly, it had little effect. Participants' performance was similar to that in Experiment 1, where no feedback was given, and where performance did not improve during the experimental session. Participants again preferred lotteries with a higher probability of winning, which is again reflected in CPT by a flat value function (see Table 2). Also the CCFs were similar to those in the previous experiment. Impulsive choices mainly followed the ML/PH strategy, whereas for slower choices also the gains were taken into account. However, this did not always lead to more optimal choices. In particular, participants had difficulties to properly integrate feedback information when confronted with similar outcome probabilities, such as for *60:40* lotteries.

Thus, outcome feedback seems not to be sufficient for generally improving choice performance. This raised questions about the type of feedback. What information might be appropriate for improving choice performance? Or are participants generally unable to learn a more optimal strategy under the present conditions? These questions were examined in the next experiment.

## Experiment 3

This experiment was similar to the previous one, except that a different type of feedback was provided. Whereas the outcome feedback in the previous experiment indicated only probabilistically whether a choice was optimal or not, the feedback in the present case indicated whether the choice was “mathematically” optimal (i.e., EV-optimal) or not. Because this feedback is not only deterministic, but also more informative than outcome feedback, we hypothesized, that it should improve performance, compared to the previous experiments.

### Methods

#### Participants

Seventeen psychology students (2 male, aged between 18 and 33 years, *M* = 21.2, *SD* = 3.5) from the Universität Konstanz, participated in the experiment, either for course credit or money (see Footnote 1). None of the participants took part in the previous two experiments.

#### Material and procedure

The procedure was the same as in Experiment 2, except that *error feedback* was given on trials on which the lottery with the lower expected value was chosen. For one second, a text was presented at the center of the screen stating that the choice was not optimal. After an optimal choice, no feedback was given and the next trial started without delay.

### Results

Choices with RTs smaller than 200 ms and larger than 3000 ms were considered as outliers and excluded from analysis (<3.7% of all data).

#### Choice behavior

The lottery with the higher winning probability was chosen more frequently in *pro-win* gambles (*M* = 78.6%, *SD* = 41.0%) than in *pro-gain* gambles (*M* = 23.2%, *SD* = 42.2%). This main effect of gamble type was significant, *F*(1, 16) = 272.99, *p* < 0.001, η_*G*^2^_ = 0.945.

#### Block by block learning

**Figure 4:**
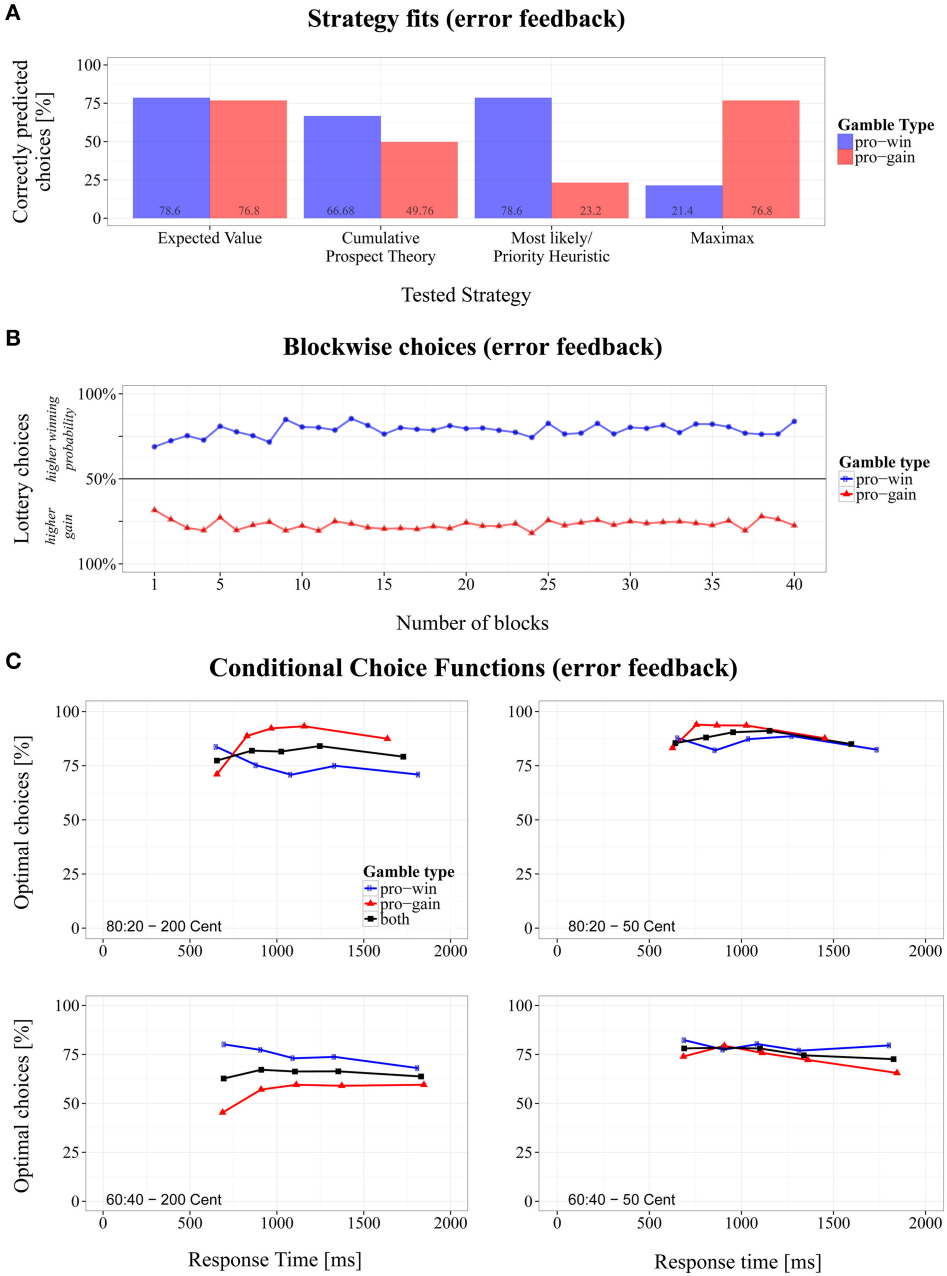
**Overview of the results for the modified Wheel of Fortune task with error feedback. (A)** Proportions of correctly predicted choices for *pro-win* and *pro-gain* gambles across different strategies, **(B)** changes of choice proportions across 40 experiment blocks, and **(C)** conditional choice functions (CCFs).

To test whether error feedback improved participants' choice during the experimental session, we computed a 2 (gamble type) × 40 (block number) repeated-measures ANOVA. The analyses revealed that block number was not involved in any significant effect. Thus, choice proportions remained steady across blocks (see also Figure 4B). However, a visual inspection suggested that there was at least a small trend of learning in the first five blocks.

#### Strategy fits

An inspection of the overall proportion of correct predictions of the tested strategies revealed that EV-based choices were of superior match (*M* = 77.7%, *SD* = 41.6%), compared to CPT (*M* = 58.5%, *SD* = 49.3%), ML/PH (*M* = 50.8%, *SD* = 50.0%), and MM (*M* = 49.2%, *SD* = 50.0%), of which only the latter two did not significantly differ from each other in a pairwise comparison (see Table 3).

By taking the gamble type into account (see Figure 4A), the predictions suggest that participants decided on the majority of trials according to EV, irrespective of whether a high gain or a high probability indicated the optimal choice, paired *t*(16) = 0.62, *p* = 0.541, Cohen's *d* = 0.21. The remaining strategies significantly differed in their fit within gamble types, paired |*t*s| > 8.55, *p*s < 0.001, and Cohen's |*d*s| > 2.78. ML/PH and CPT fared better in predicting *pro-win* gambles, whereas the strategy fit for MM was better in *pro-gain* gambles. Note that ML/PH and MM predicted performance well only in gambles for which these strategies were optimal. Due to the design of gambles types, however, the overall fit was the same.

#### Conditional choices

A 2 (probabilities) × 2 (gain differences) repeated-measures ANOVA of slopes derived from CCFs yielded a significant main effect of gain difference, *F*(1, 16) = 5.63, *p* < 0.05, η_*G*^2^_ = 0.050, (*b*_50_ = −0.004 and *b*_200_ = 0.001). When confronted with lotteries whose gains differed by 200 Cent, the choice proportions remained similar with an increasing RT. With a gain difference of 50 Cent, the proportion of optimal choices slightly decreased. Whereas in Experiments 1 and 2, the mean RTs of the fastest responses were about 400 ms, they increased in the present experiment to about 600 ms. No other significant effects were found. However, as can be seen in Figure 4C, even fast responses were already at a relatively high level of EV-optimality.

#### Comparison with Experiment 2

We compared the mean slope and intercept with those in the previous experiment. The difference between the mean slopes (Exp. 2: 0.011; Exp. 3: −0.001) was significant, paired *t*(33.3) = 3.912, *p* < 0.001, Cohen's *d* = 1.31, as was the difference between the intercepts (Exp. 2: 49.1: Exp. 3: 79.3), paired *t*(22.8) = −8.56, *p* < 0.001, Cohen's *d* = −2.86.

### Discussion

The present results demonstrate that participants can indeed improve their choice performance if they are provided with a highly informative feedback. By signaling whether their choice was EV-optimal or not, choices became more EV-optimal, compared to no feedback (Experiment1), or outcome feedback (Experiment 2). Comparing the predictions of the different strategies to the data revealed that the EV strategy accounted better for the performance than the CPT. This is surprising, because the EV strategy is a special case of the CPT. A possible explanation could be that the fit procedure did not work well. Since performance was more EV-optimal than in the previous experiments, fitting the CPT might this time suffer from an over-parameterization.

The type of feedback also affected the CCFs. This time they were relatively flat, but started at a more optimal level, also for *pro-gain* gambles. Thus, it seems that the participants integrated probabilities and gains also for relatively fast choices. However, the fastest choices were generally slower, compared to the previous experiments. Finally, our data indicated that the participants learned rather quickly to improve their performance if error feedback was provided.

## General discussion

The various strategies that have been proposed to explain how people deal with risky-choice problems differ, among others, in the amount of information that is considered. Whereas some strategies assume that all relevant information is taken into account for a decision (e.g., von Neumann and Morgenstern, 1944; Kahneman and Tversky, 1979), others suppose that only some of that information is utilized (e.g., Brandstätter et al., 2006; Gigerenzer and Gaissmaier, 2011). The extent to which either is the case varies strongly between individuals (e.g., Venkatraman et al., 2009), but also depending on whether choices were based on *description* or *experience* (e.g., Glöckner et al., 2012). However, it is largely unknown how much information is considered when participants are fully informed about all probabilities and outcomes, and when they have multiple trials on which they can evaluate their utilization of the available information through feedback and possibly adjust their choice on subsequent trials.

To investigate choice behavior in such interesting but rarely studied situations, we conducted a series of three experiments in which participants had repeatedly to choose between two fully described lotteries. A specific question was whether participants based their decisions on the winning probabilities, on the possible gains, or on both. Moreover, we wanted to examine to what extent the type and amount of information used for deciding changes with experience, and whether it depends on the nature of the provided feedback. Finally, we were interested in the dynamics of decision making within a given trial.

For answering these question, we used gambles in which either the lottery with the higher winning probability (*pro-gain*) or the one with the larger potential gain (*pro-win*) coincided with the EV-optimal choice. From other studies it is well known that decision makers have the strong tendency to prefer the option with the higher probability, at least if losses are not possible (e.g., Tversky and Kahneman, 1981; Fiedler and Unkelbach, 2011). In our experiments, this probability dominance would have been optimal for *pro-win* gambles, but suboptimal for *pro-gain* gambles. Thus, to achieve a proportion of optimal choices larger than 50%, probabilities and gains would have to be integrated in some way. As the results of our first experiment indicate, such an integration hardly took place. The participants mostly chose the lottery with the higher winning probability, and largely ignored the potential gains. This was also confirmed by comparing the predictions of different decision models with the observed choice behavior. The best fit was obtained with the *Most-Likely* heuristic and with a CPT model, where probability-dominance was reflected by flat value functions.

Beyond the common inspection of strategy fits, a far more detailed analysis of information integration that also accounts for the dynamics of the decision process was achieved by examining conditional choice functions (CCFs). Instead of a static preference for lotteries with a higher winning probability, we observed that this preference decreased with response time (RT). Moreover, performance was improved for slow relative to fast decisions. This not only suggests that participants applied different strategies across trials, but also that more information was considered for slower decisions (see also Dambacher et al., unpublished manuscript). A possible scenario is provided by the dual-process idea (e.g., Hübner et al., 2010; Mukherjee, 2010). It is conceivable that suboptimal but fast and automatic heuristic processes (e.g., *Most-Likely*, or *Maximax*) competed with more optimal but slow and controlled processes (e.g., CPT) for execution. On some trials the participants impulsively chose the lottery with the higher winning probability, whereas on other, presumably if they managed to suppress their impulsive choice tendency, the possible gains were also taken into account, at least to some extent. This is in line with the idea that risky decision making is based on a toolbox of strategies (e.g., Payne et al., 1993; Gigerenzer and Selten, 2001). An alternative explanation for the observed deviations is proposed by models that assume that participants intend to use a single strategy but make erroneous choices (e.g., Bröder and Schiffer, 2003). Yet, such error models might not be able to account for systematic variations with RT.

Nevertheless, despite the fact that the decisions were slightly improved for slower decisions, the overall performance was relatively poor and far from EV-optimal. Moreover, information usage did not change during the experimental session. This might not be surprising, given that there was no feedback. Therefore, to see whether performance improves if some feedback is provided, we conducted Experiment 2, where each gamble was played by the computer and the outcome displayed to the participants. Unexpectedly, this feedback had no substantial effect. Performance was similar as in Experiment 1. Does this result indicate that persons are generally unable to learn optimal decision making? Or is outcome feedback simply useless for learning? To answer these questions we ran Experiment 3, where a normative error feedback was provided. That is, if a decision was not EV-optimal, the participants were informed that their performance was suboptimal. As a result, decision performance improved substantially. Obviously, this type of feedback motivated the participants to base their decisions not only on probability information, but to also consider the possible gains. That this was indeed the case is supported by the fact that the decision times were generally increased. Moreover, this time choice proportions were rather constant with RT.

How can the different effectivity of the two feedback types be explained? Outcome feedback might be rather useless, because the provided information is probabilistic and, therefore, ambiguous. In particular for *60:40* lotteries, a positive outcome frequently signals a “good choice,” even when the choice was not EV-optimal. In other studies, however, outcome feedback was not ignored. Yechiam and Busemeyer (2006), for example, observed reduced choice proportions for risky, suboptimal options. In their experiment in the loss domain, very small probabilities certainly provided less ambiguous feedback (sample gamble: lose 8 [300] cents with a probability of 5%, otherwise lose 2 [1] cents; competing lottery in brackets). Larger differences between the probabilities, or rare events can provide a better opportunity for optimally reevaluating lotteries. Thus, it is reasonable to assume that the composition of choice problems is likely to interact with the applicability of outcome feedback.

Error feedback, on the other hand, provides a deterministic and consistent signal of how to choose optimally. Accordingly, participants quickly learn to combine the available information in a rather optimal way for their decisions. However, feedback might not have had the same effect for each participant. Research has already shown that individuals differ with respect to learning from feedback (e.g., Schonberg et al., 2007). In our experiments, individual differences were more apparent for outcome feedback. When we exploratively looked for individual choice behavior, we found, for example, that one participant permanently chose the lottery with the higher gain, whereas another predominantly chose randomly. The variation between individual CCFs was also relatively large. However, choice behavior was relatively similar between participants when error feedback was provided. This underpins the effectiveness of error feedback in facilitating information integration and shaping choice behavior.

Taken together, our results show that participants mainly use only some of the available information for decision making, even if outcome feedback is provided. Specifically, there is a strong probability dominance, i.e., mostly the lottery with the higher winning probability is chosen. This is especially true for fast decisions. Only if normative error feedback is provided, participants learn to take also the possible gains into account. It is open, however, how general these conclusions are, because we imposed several constraints on our gambles. One consequence is, for example, that the EVs in the different gamble types were not balanced, which could have influenced information processing (e.g., Ayal and Hochman, 2009). Whether further studies with different gambles but a similar setting will find comparable results, or how long the learning effects from error feedback will last, has yet to be shown.

### Conflict of interest statement

The authors declare that the research was conducted in the absence of any commercial or financial relationships that could be construed as a potential conflict of interest.

## Footnotes

^1^The experiment was performed in accordance with the ethical standards laid down in the 1964 Declaration of Helsinki and its later amendments. In agreement with the ethics and safety guidelines at the Universität Konstanz, we obtained a verbal informed consent statement from all individuals prior to their participation in the study. Potential participants were informed of their right to abstain from participation in the study or to withdraw consent to participate at any time without reprisal.

^2^We applied the three rules formally described by Glöckner and Betsch (2008). As the minimum gains of both lotteries were zero and did not differ, Step 1 had to be skipped. In Step 2, the probabilities of the minimum gains had to be compared. For each of the lotteries, the differences of the minimum probabilities *p*_*min*(*B*)_ − *p*_*min*(*A*)_ exceeded the aspiration level of 0.10 (0.80 − 0.20 = 0.60 or 0.60 − 0.40 = 0.20), thus Lottery A had to be chosen. As Step 2 applied to all lotteries, Step 3 was not considered.

^3^For some combinations of lottery values, probabilities and parameter sets, the Luce rule component *e*^φ·*V*(*A*)^ can yield infinite values. We solved the problem by setting the choice predictions of respective lotteries to 1. Additionally, we added a penalty to the goodness-of-fit measure *G*^2^, in order to avoid the optimization routine to further explore an inappropriate parameter space.

^4^The L-BFG-S method within the *optim*-function allows the setting of independent constraints for each parameter.

^5^Detailed analyses for the comparisons of *pro-win* and *pro-gain* gambles are available from the authors upon request.
